# P237: Nosocomial infections and infectious agents determined in geriatric patients

**DOI:** 10.1186/2047-2994-2-S1-P237

**Published:** 2013-06-20

**Authors:** H Gül, A Karakaş, C Artuk, S Aytemiz, G Mert, CP Eyigün

**Affiliations:** 1 Infectious Diseases and Clinical Microbiology, Gulhane Military Hospital, Ankara, Turkey; 2 Infection Control, Infection Control, Ankara, Turkey

## Introduction

Parallel to the increase in the elderly population, various health policies including materialistic and moral policies have been developed for this patient group worldwide, especially in developed countries.

## Objectives

The aim of this study was to determine the nosocomial infections that emerge during the hospitalization of the elderly population, the risk factors for these infections and the mortality rates.

## Methods

The frequencies, types, agent distributions and the emerging periods of nosocomial infections, and the risk factors for these infections in patients aged 65 and over, who had been treated and followed-up in GMMA in 2011, were retrospectively evaluated.

## Results

610 nosocomial infections were observed in 400 patients in our hospital in 2011. 188 of these patients were geriatric patients and 279 nosocomial infections were determined among them. 77 of the patients were female and 111 were male. The mean age was 76,99±7,74. 52% of the patients were followed-up in intensive care units, whereas 48% were followed-up in the clinics. The emergency internal medicine intensive care unit was the clinic with the highest number of geriatric patients, which was 45 (22,9%). Eighty-five of 400 patients (21,3%), who were all geriatric patients with determined nosocomial infection, died. The types of nosocomial infection were urinary system infection in 45,8%, bloodstream infection in 32,9%, pneumonia in 8,6% and surgical site infection in 7,8%. A total of 289 microorganisms were isolated from 279 nosocomial infections. The infectious agents were *Escherichia coli* in 20,1%, *Klebsiella pneumoniae* in 13,1%, *Acinetobacter baumannii* in 10,7%, *Pseudomonas aeruginosa* in 5,2% and *Candida* spp in 4,8%. The development of nosocomial infections was observed to be a mean of 20,88±16,83 days as of the day of hospitalization. A total of 541 interventions were performed on 188 patients with nosocomial infection. These interventions were urinary catheter installation, peripheral and central venous catheter installation, mechanical ventilation and endotracheal intubation.

## Conclusion

The mortality-survival rates in nosocomial infections of geriatric patients is 50%>50%, and the risk factors were determined to be the interventional procedures, prolonged hospitalization and previous nosocomial infections.

## Disclosure of interest

None declared

